# MicroRNA-130a Contributes to Type-2 Classical DC-activation in Sjögren's Syndrome by Targeting Mitogen- and Stress-Activated Protein Kinase-1

**DOI:** 10.3389/fimmu.2019.01335

**Published:** 2019-06-20

**Authors:** Ana P. Lopes, Joel A. G. van Roon, Sofie L. M. Blokland, Maojie Wang, Eleni Chouri, Sarita A. Y. Hartgring, Kim M. G. van der Wurff-Jacobs, Aike A. Kruize, Boudewijn M. T. Burgering, Marzia Rossato, Timothy R. D. J. Radstake, Maarten R. Hillen

**Affiliations:** ^1^Laboratory of Translational Immunology, University Medical Center Utrecht, Utrecht University, Utrecht, Netherlands; ^2^Department of Rheumatology & Clinical Immunology, University Medical Center Utrecht, Utrecht University, Utrecht, Netherlands; ^3^Department of Molecular Cancer Research, Center Molecular Medicine, Oncode Institute, University Medical Center Utrecht, Utrecht University, Utrecht, Netherlands; ^4^Department of Biotechnology, University of Verona, Verona, Italy

**Keywords:** primary Sjögren's syndrome, microRNA, miR-130a, conventional dendritic cell, BDCA1+ myeloid DC, MSK1

## Abstract

**Objectives:** Considering the critical role of microRNAs (miRNAs) in regulation of cell activation, we investigated their role in circulating type-2 conventional dendritic cells (cDC2s) of patients with primary Sjögren's syndrome (pSS) compared to healthy controls (HC).

**Methods:** CD1c-expressing cDC2s were isolated from peripheral blood. A discovery cohort (15 pSS, 6 HC) was used to screen the expression of 758 miRNAs and a replication cohort (15 pSS, 11 HC) was used to confirm differential expression of 18 identified targets. Novel targets for two replicated miRNAs were identified by SILAC in HEK-293T cells and validated in primary cDC2s. Differences in cytokine production between pSS and HC cDC2s were evaluated by intracellular flow-cytometry. cDC2s were cultured in the presence of MSK1-inhibitors to investigate their effect on cytokine production.

**Results:** Expression of miR-130a and miR-708 was significantly decreased in cDC2s from pSS patients compared to HC in both cohorts, and both miRNAs were downregulated upon stimulation via endosomal TLRs. Upstream mediator of cytokine production MSK1 was identified as a novel target of miR-130a and overexpression of miR-130a reduced MSK1 expression in cDC2s. pSS cDC2s showed higher MSK1 expression and an increased fraction of IL-12 and TNF-α-producing cells. MSK1-inhibition reduced cDC2 activation and production of IL-12, TNF-α, and IL-6.

**Conclusions:** The decreased expression of miR-130a and miR-708 in pSS cDC2s seems to reflect cell activation. miR-130a targets MSK1, which regulates pro-inflammatory cytokine production, and we provide proof-of-concept for MSK1-inhibition as a therapeutic avenue to impede cDC2 activity in pSS.

## Introduction

Primary Sjögren's syndrome (pSS) is an autoimmune disease characterized by keratoconjunctivitis sicca, xerostomia, and lymphocytic infiltration of salivary and lacrimal glands (1, 2). pSS is associated with multiple factors such as genetic predisposition and environmental factors including viral infection (3). Although the cause of pSS remains poorly understood, the contribution of the immune system is evident. Activated autoreactive B cells and T cells as well as increased levels of pro-inflammatory cytokines drive chronic inflammation of the exocrine glands, associated with loss of function (4).

Conventional dendritic cells (cDCs) are potent antigen-presenting cells with an important role in the initiation and control of immune responses, mainly due to their superior ability to take up and present antigens to T cells. cDCs can be divided into two phenotypically and functionally distinct subsets defined by their expression of CD141 (cDC1s) and CD1c (cDC2s). cDC2s are the most predominant in human blood, tissues and lymphoid organs (5). They produce a variety of cytokines (e.g., IL-12, IL-6, and TNF-α) and chemokines (e.g., CXCL8, CCL3, CCL4, CCL5, and CXCL10) (6) and present antigen to potently activate T cells (7, 8). The primary target cells of cDC2s, CD4 T cells, are thought to play a crucial role in pSS immunopathology (2, 4) and increased numbers of DCs are present in salivary glands of pSS patients (9, 10). As such, cDC2s are suspected to play an important role in driving salivary gland inflammation (11, 12), however their molecular regulation has not yet been studied in pSS.

MicroRNAs (miRNAs) are small non-coding RNAs that post-transcriptionally regulate gene expression by inducing cleavage of their target mRNA or preventing translation into protein (13). miRNAs are critical regulators of numerous biological processes, such as cell proliferation and differentiation, metabolism, and cell activation (14, 15). In pSS, several studies showed that miRNA expression is dysregulated in peripheral blood mononuclear cells (PBMCs), purified immune cells, and salivary gland tissue (16–18). Changes in miRNA expression were associated with the presence of autoantibodies, local inflammation, and the production of pro-inflammatory cytokines (19–22). To unravel the molecular mechanisms that regulate cDC2 function in patients with pSS, we investigated the miRNA profile of purified circulating cDC2s from patients with pSS and identified two miRNAs that are expressed at a lower level in pSS in two independent cohorts. In addition, we establish mitogen- and stress-activated protein kinase-1 (MSK1) as a novel endogenous target of miR-130a in primary cDC2s and provide proof-of-concept for targeting this kinase to inhibit the production of pro-inflammatory cytokines by cDC2s.

## Materials and Methods

### Patients and Controls

All pSS patients met the 2002 AECG classification criteria (23). A group of healthy controls (HC) was included as a control group. For identification of differentially expressed miRNAs, two independent cohorts of patients and controls (discovery and replication) were established. An independent set of HC and pSS donors was included for follow-up experiments (Table 1). The study was approved by the medical ethics committee of the University Medical Center Utrecht (METC no. 13-697). All patients gave their written informed consent in accordance with the declaration of Helsinki.

**Table 1:** Characteristics of the patients and controls enrolled in the study.

	**miRNA profiling**				**MSK1 analyses**		**Intracellular staining**	
	**Discovery phase (*****n*** **=** **21)**		**Replication phase (*****n*** **=** **26)**		**(*****n*** **=** **41)**		**(*****n*** **=** **25)**	
	**HC**	**pSS**	**HC**	**pSS**	**HC**	**pSS**	**HC**	**pSS**
N (M/F)	6 [0/6]	15 [3/12]	11 [1/10]	15 [1/14]	16 [0/16]	25 [3/22]	12 [0/12]	13 [0/13]
Age (yr.)	56 [54-67]	53 [29-77]	50 [26-55]	55 [26-69]	57 [45-67]	58 [34-77]	58 [26-63]	61 [22-81]
LFS (foci/4 mm^2^)	-	1.7 [1.0–4.0]	-	2.0 [1.0–4.0]	-	2.0 [1.0–5.0]	-	3.3 [1.0–6.4]
ESSDAI	-	2.0 [0.0–19]	-	5.0 [0.0–13]	-	3.0 [0.0–19]	-	5.0 [0.0–13]
ESSPRI	-	3.3 [1.8–8.8]	-	5.3 [1.0–8.0]	-	4.9 [1.3–8.8]	-	6.0 [2.0–9.0]
Schirmer (mm/5 min)	-	4.5 [0.5–25]	-	15 [0.5–30]	-	5.5 [0.0–28]	-	5.0 [0.0–24]
ANA (no. positive [%])	-	10 [67%]	-	13 [93%]	-	18 [75%]	-	13 [100%]
SSA (no. positive [%])	-	8 [53%]	-	12 [80%]	-	17 [68%]	-	13 [100%]
SSB (no. positive [%])	-	3 [20%]	-	9 [60%]	-	11 [44%]	-	11 [85%]
RF (no. positive [%])	-	4 [31%]	-	7 [64%]	-	9 [43%]	-	7 [78%]
Serum IgG (g/L)	-	14 [8.3–30]	-	18 [9.3–33]	-	14 [5.6–42]	-	12 [8.4–22]
ESR (mm/hour)	-	9 [5.0–30]	-	17 [4.0–77]	-	14 [4.0–43]	-	14 [2.0–27]
CRP (mg/L)	-	1.0 [0.0–8.0]	-	1.0 [0.0–49]	-	2.0 [0.0–13]	-	1.7 [0.6–22]
C3 (g/L)	-	1.1 [0.8–1.3]	-	1.0 [0.5–1.3]	-	1.1 [0.8–1.6]	-	0.9 [0.9–1.1]
C4 (g/L)	-	0.3 [0.1–0.3]	-	0.2 [0.1–0.3]	-	0.3 [0.1–0.4]	-	0.2 [0.1–0.3]
Not treated (no. [%])	-	12 [80%]	-	9 [60%]	-	19 [76%]	-	5 [38%]
Only HCQ (no. [%])	-	1 [7%]	-	2 [13%]	-	2 [8%]	-	5 [38%]
Other (no. [%])	-	2 [13%]	-	4 [27%]	-	4 [16%]	-	3 [23%]

*HC, healthy control; pSS, primary Sjögren's syndrome; LFS, lymphocyte focus score; ESSDAI, EULAR Sjögren's syndrome disease activity index; ESSPRI, EULAR Sjögren's syndrome patient reported index; ANA, anti-nuclear antibodies; SSA, anti-SSA/Ro; SSB, anti-SSB/La; RF, rheumatoid factor; ESR, erythrocyte sedimentation rate; CRP, C-reactive protein, HCQ, hydroxychloroquine. Other treatment group includes azathioprine, alone (n = 2) or in combination with prednisone (n = 2); mesalazine (n = 2); prednisone, alone (n = 4) or in combination with HCQ (2); methotrexate (n = 1). Values are median [range] unless stated otherwise*.

### cDC2 Isolation

cDC2s were isolated from peripheral blood or buffy coats (Sanquin) by magnetic-activated cell sorting using CD1c (BDCA-1)+ Dendritic Cell Isolation Kit (Miltenyi Biotec) according to the manufacturer's instructions. To confirm consistent purity of isolated cDC2s, the proportion of CD19-BDCA1+ cells within the isolated fraction was measured using fluorescence activated cell sorting (FACS). The purity of the isolated samples was (median [interquartile range]) 94% (88–97%), and there were no significant differences in cell purity between any of the groups.

### RNA Isolation

For mRNA and miRNA studies, cells were lysed in RLTPlus buffer (Qiagen) and total RNA was purified using AllPrep DNA/RNA/miRNA Universal Kit (Qiagen) according to the manufacturer's instructions. RNA concentration was assessed with Qubit RNA Kit (Thermo Fisher Scientific).

### miRNA Quantification in Discovery and Replication Cohorts

Identification of differentially expressed miRNAs was carried out in two independent phases. First, expression of 758 miRNAs was screened in MACS-isolated CD1c-expressing cDC2s from the donors included in the discovery cohort using the OpenArray platform as previously described (24, 25) (discovery phase; Supplementary Figure 1). miRNAs with a poor amplification score (<1.24) were excluded from the analysis and low expressed miRNAs (CT>27) were set at 27. Data were normalized using the global mean normalization approach. miRNAs with a fold change (FC) difference of >1.5 at a nominal *p*-value < 0.05 between the groups were considered to be differentially expressed. Selection of miRNAs to be measured in the replication cohort was based on the expression level and quality of the amplification (Supplementary Figure 1). Consistent differential expression was confirmed for 18 selected miRNAs in the replication cohort using a custom array (Thermo Fisher Scientific). The array included the 18 selected miRNAs and 4 reference small non-coding RNAs (sncRNAs) (miR-17, miR-191, RNU48, U6-snRNA) that all showed good abundance and stable expression between patients and controls (replication phase; Supplementary Figure 1). Data were analyzed according to the comparative threshold cycle (CT) method and the expression of each sample was normalized by the mean expression of the four reference sncRNAs. Relative expression was depicted as FC compared to one HC sample, which was set at 1. Replication was considered successful if the direction of the difference (i.e., up/downregulation) was identical to what was observed in the discovery cohort and the difference was significant at a nominal *p*-value < 0.05.

### cDC2 Stimulation

cDC2s were cultured in RPMI glutamax (Thermo Fisher Scientific) supplemented with 10% heat-inactivated fetal calf serum (FCS) (Biowest Riverside) and 1% penicillin/streptomycin (Thermo Fisher Scientific). cDC2s were cultured at a concentration of 0.5 × 10^6^ cells/mL in a 96-well round-bottom plate. Cells were left unstimulated or were stimulated for 24 h either with 25 μg/mL of toll-like receptor (TLR) 3 ligand Poly(I:C) or with 1 μg/mL of TLR7/8 ligand R848 (both InvivoGen).

### miRNA Quantification in Cultured Cells

Analysis of miRNA expression in cultured cells was performed using TaqMan miRNA Human Assays hsa-miR-130a-3p (ID 000454), hsa-miR-708-5p (ID 002341) and RNU44 (ID 001094) (Thermo Fisher Scientific). cDNA was prepared by using the TaqMan MicroRNA Reverse Transcription Kit according to the manufacturer's instructions (Thermo Fisher Scientific). miRNA levels were quantified using TaqMan fast advance master mix and miRNA-specific primers from the TaqMan miRNA assays on the Quantstudio 12k Real-Time PCR system (Thermo Fisher Scientific). Relative miRNA expression was calculated after normalization by RNU44, which was stably expressed across groups and conditions, using the comparative CT method. The relative fold change (FC) of each sample was calculated in comparison with the unstimulated or the non-targeting miRNA control (SCR) transfected condition where appropriate.

### miRNA Transfection of HEK-293T Cells and Primary cDC2s

HEK-293T cells were cultured in DMEM (Thermo Fisher Scientific) with 10% FCS (Biowest) and 1% penicillin/streptomycin (Thermo Fisher Scientific). 24 h prior to transfection, HEK-293T cells were plated in a 6-well plate at a concentration of 0.5 × 10^5^ cells/mL. On the day of transfection, the medium was replaced and cells were transfected with miR-130a mimic or with non-targeting miRNA control (SCR) (Thermo Fisher Scientific) at a final concentration of 30 nM, together with lipofectamine RNAiMAX and Opti-MEM (both Thermo Fisher Scientific) for 48 h.

cDC2s were isolated from buffy coats and plated at a density of 1.0 × 10^6^/mL in a 12-well plate and rested for 6 h. Then, cells were transfected with miR-130a mimic or SCR at a final concentration of 30 nM together with lipofectamine 2,000, Opti-MEM, and PLUS Reagent (all from Thermo Fisher Scientific). 18 h post transfection, cells were washed and seeded at the same cell density and kept in culture for an additional 24 h.

Transfection efficiency was confirmed in HEK-293T by transfection of cells with a fluorescently labeled-SCR (Thermo Fisher Scientific) as described above (Supplementary Figure 2. 48 h after transfection, cells were washed and the percentage of positive cells was assessed by FACS. Additionally, the expression of miR-130a was measured in both SCR and miRNA-transfected conditions for both cell types to confirm miRNA overexpression.

### Protein Extraction and Immunoblotting

Cells were lysed in Laemmli's buffer and protein content was quantified with a BCA Protein Assay Kit (Pierce). Proteins were separated on 4–12% Bis-Tris SDS NuPAGE gels (Thermo Fisher Scientific) and transferred to polyvinylidene difluoride membranes (Bio-Rad). The membranes were blocked in 5% non-fat milk (Bio-Rad) in TBST and probed overnight at 4°C with antibodies recognizing mitogen- and stress-activated protein kinase-1 (MSK1) (#3489, rabbit anti-human, Cell Signaling Technology) and histone 3 (H3) (#9715, rabbit anti-human, Cell Signaling Technology). After washing, membranes were incubated with horseradish peroxidase (HRP)-conjugated swine anti-rabbit antibody (Agilent Technologies), and protein visualization was performed using a ChemiDoc MP system (Bio-Rad).

### Intracellular Cytokine Measurement in cDC2

To evaluate differences in cytokine production between patients and controls after *in vitro* TLR stimulation, whole blood was diluted 1:1 in RPMI-1640 medium (Thermo Fisher Scientific) with 1% L-glutamine (Thermo Fisher Scientific) and stimulated with TLR4 ligand LPS (25 μg/mL, Sigma). 1 h after stimulation, 10 μg/mL of Brefeldin A (Sigma) was added and incubated for 5 h. Cells were then stained with anti-BDCA-1 APC (L161, Thermo Fisher Scientific), anti-CD19 BV510 (HIB19, BioLegend), anti-HLA-DR BV605 (G46-6, BD Biosciences) and anti-CD14 BV785 (M5E2, BioLegend). After washing, fixation and permeabilization with FIX&PERM (Thermo Fisher Scientific) according to manufacturer's instructions, cells were stained with anti-IL-6 AF700 (MQ-13A5, Thermo Fisher Scientific), anti-IL-12 FITC (C11.5, BD Biosciences), anti-IL-8 PerCP-Cy5.5 (BH0814, Sony Biotechnology) and anti-TNF-α BV421 (MAb11, BioLegend). Data acquisition was performed using a BD LSRFortessa (BD Biosciences) and data were analyzed using FlowJo software (Tree Star).

### cDC2 Stimulation and Exposure to MSK1 Inhibitors

cDC2s isolated from buffy coats were plated at a density of 0.5 × 10^6^/mL in a 96-well round-bottom plate. Cells were left unstimulated or were treated with MSK1 inhibitors [H89, 10 μM (Bio-Techne); SB 747651A, 10 μM (Bio-Techne); or Ro 31-8220, 5 μM (Sigma)] for 1 h. Then, cells were stimulated with TLR4L at a final concentration of 100 ng/ml. After 6 h, supernatants were stored and cells were lysed for RNA extraction or processed for flow cytometry.

After harvesting, cells were washed in Annexin V Binding Buffer and stained with Annexin V–APC, 7-AAD–PerCP (all from BD Biosciences), anti-CD80–PE (L307.4, BD Biosciences), anti-CD83–FITC (HB15a, Beckman Coulter) and anti-CD86–PB (IT2.2, Sony Biotechnology). Data acquisition was performed using a FACSCanto II flow cytometer (BD Bioscience) and data were analyzed using FlowJo software (Tree Star). The percentage of viable cells after stimulation was measured as the proportion of Annexin V/7AAD double negative cells (Supplementary Figure 3). The expression of co-stimulatory molecules given by the mean fluorescent intensity was evaluated within the viable cells.

### Statistics

Differences in miRNA expression between pSS patients and HCs in the discovery cohort were analyzed using Thermofisher Cloud software. For analysis of the replication cohort data, differences in miRNA expression between pSS and HC were assessed using the Mann-Whitney U test (two-sided). For unsupervised hierarchical clustering, Euclidean distance and Ward's linkage method were used on the miRNA FC using MetaboAnalyst online software (https://www.metaboanalyst.ca/). Wilcoxon signed-rank test was used for paired comparisons in *in vitro* cultures. Statistical analyses were performed using SPSS v20 (IBM) and Graphpad Prism (GraphPad Software). Differences were considered to be statistically significant at *p* < 0.05.

Detailed descriptions of stable isotope labeling of amino acids in cell culture (SILAC), selection of *in silico* predicted miRNA targets, quantitative real-time PCR and cytokine analysis are provided in the Online Supplementary Methods.

## Results

### Expression of miR-130a and miR-708 Is Consistently Decreased in cDC2s From pSS Patients, Associated With Cell Activation

**Figure 1:**
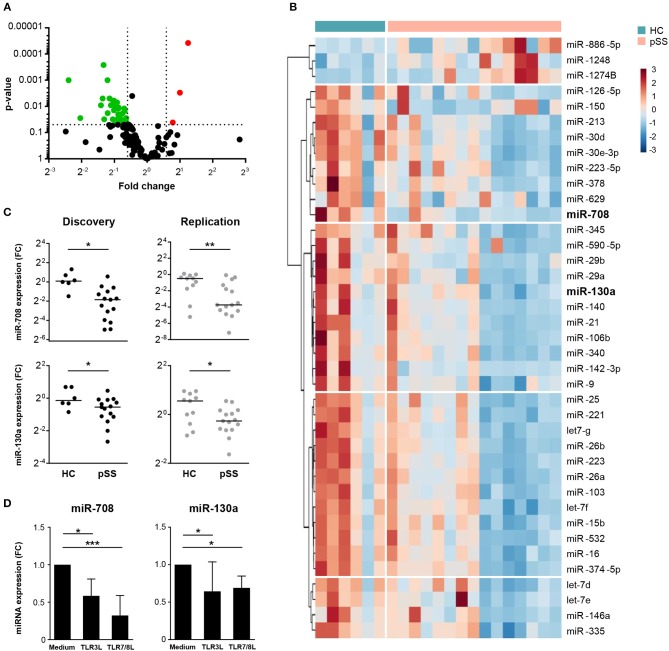
Expression of miR-708 and miR-130a is decreased in pSS patients. Volcano plot displays the relationship between fold change (x-axis) and the statistical significance (y-axis) for the compared groups (pSS patients vs. HC) **(A)**. Hierarchical clustering of the 39 differentially-expressed miRNAs between pSS patients and HC using Euclidean distance and Ward's method **(B)**. miR-708 and miR-130a expression was consistently downregulated in pSS patients compared to HC in both cohorts **(C)**. cDC2s stimulated with TLR3 (25 μg/mL) and TLR7/8 (1 μg/mL) ligands for 24 h showed a reduced expression of miR-708 and miR-130a measured by qPCR compared to medium control **(D)**. Medians ± IQR are shown *p < 0.05, **p < 0.01, and ***p < 0.001, respectively.

Using two independent cohorts of patients and controls (Table 1) we identified differentially expressed miRNAs in cDC2s from pSS patients compared to HC. In the discovery phase, we screened the expression of 758 miRNAs, of which 143 were expressed in cDC2s (Supplementary Figure 1). Of these, 39 were differentially expressed in pSS patients compared to HC (0.67< *FC* >1.5; *p* < 0.05) (Figures 1A,B and Supplementary Table 1). Of the 39 identified miRNAs, we selected 18 miRNAs for quantification in the replication cohort based on their expression level and the quality of amplification in the array (Supplementary Figure 1). The expression of miR-708 and miR-130a was consistently decreased in pSS patients compared to HC in two independent cohorts (biological replication) (Figure 1C).

Immune cells are known to modulate their miRNA expression upon activation (26). To investigate whether cDC2 activation is associated with downregulation of miR-708 and miR-130a in pSS, cDC2s from HCs were stimulated with ligands for TLR3 and TLR7/8, which are endosomal nucleic acid receptors that are relevant in pSS pathophysiology (27, 28). We observed that upon cell activation with either TLR ligand the expression of both miR-708 and miR-130a was decreased when compared with the unstimulated condition (Figure 1D). This supports the notion that the decreased expression of these miRNAs in pSS reflects cDC2 activation.

### MSK1 Is a Novel Target of miR-130a

**Figure 2:**
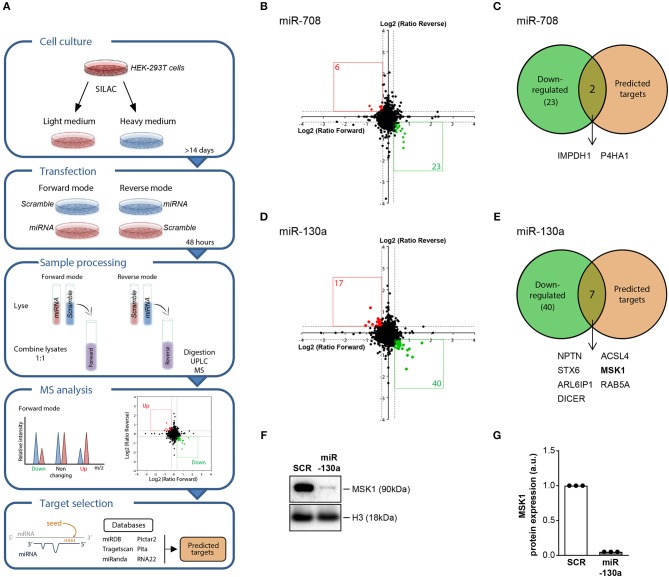
SILAC-based proteomics approach identifies MSK1 as novel target of miR-130a. HEK-293T cells were cultured in light medium or in heavy medium and transfected with either miRNA or with non-targeting miRNA control (SCR) for 48 h. After transfection, light and heavy medium-cultured cells were lysed and combined for mass spectrometry analysis. The intensity of the peak ratios between heavy and light peptides reflects changes in protein production. *In silico* predicted and experimentally validated targets of the miRNAs were retrieved from six publicly available databases **(A)**. Proteins that were downregulated after miRNA-708 or miR-130a overexpression, depicted in green **(B,D)**, were selected and compared to the selected targets for each miRNA **(C,E)**. Of the 7 proteins that were both downregulated upon miRNA-130a overexpression and contained a seed-region, MSK1 was selected for further analysis. The downregulation of MSK1 in HEK-293T cells upon miRNA-130a overexpression was confirmed in 3 independent experiments by western blot **(F)** (representative of 3 independent experiments) and protein amount was quantified by densitometry in relation to the paired sample transfected with the SCR control, which was normalized to 1 **(G)**.

To investigate the regulatory effect of the replicated miRNAs on gene expression at the protein level, we used stable isotope labeling of amino acids in cell culture (SILAC). This proteomic approach allows the identification of miRNA targets by comparing the protein production in cells transfected with a miRNA mimic and non-targeting control (Figure 2A). For miR-708, overexpression resulted in downregulation of 23 proteins (Figure 2B), of which 2 represented *in silico*-predicted targets: inosine-5′-monophosphate dehydrogenase 1 (IMPDH1) and prolyl 4-hydroxylase subunit alpha 1 (P4HA1) (Figure 2C and Supplementary Table 2). Upon overexpression of miR-130a, the expression of 40 proteins was downregulated (Figure 2D). 7 of these proteins represented *in silico*-predicted targets (Figure 2E and Supplementary Table 2). One of the identified miR-130a targets, MSK1, is an important mediator upstream of NF-κB that controls the production of pro-inflammatory cytokines by cDC2s (29, 30). As such, we selected MSK1 as a target to investigate in follow-up experiments. For this, we first sought to confirm MSK1 regulation by miR-130a at the protein level in HEK-293T cells using western blot. Indeed, in three independent experiments MSK1 was downregulated upon miR-130a overexpression (Figures 2F,G).

### MSK1 Is an Endogenous Target of miR-130a in Primary cDC2 and MSK1 Expression Is Increased in pSS Patients

**Figure 3:**
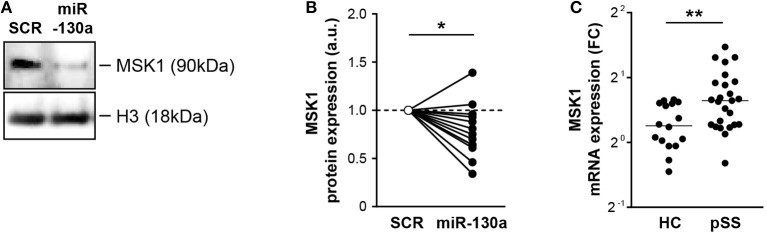
miR-130a regulates MSK1 in cDC2s and MSK1 expression is increased in pSS cDC2s. Downregulation of MSK1 upon miR-130a overexpression in primary cDC2s was confirmed at the protein level by western blot **(A)** (representative of 13 independent experiments). Protein amount was quantified by densitometry in relation to the paired sample transfected with the SCR control, which was normalized to 1 **(B)**. MSK1 mRNA expression was measured by qRT-PCR and calculated as fold change (FC) in comparison with the ΔCt mean of the HC group **(C)**. Medians are shown. *p < 0.05 and **p < 0.01, respectively.

To determine whether miRNA-130a also regulated MSK1 protein expression in cDC2s, miR-130a was overexpressed in purified cDC2s from HCs. In-line with the data from HEK-293T cells, miR-130a overexpression resulted in significantly decreased expression of MSK1 (Figures 3A,B). Following our observation that miRNA-130a was decreased in cDC2s from pSS patients, we investigated whether MSK1 expression was also dysregulated in these cells. Further corroborating the regulation of MSK1 by miR-130a, the expression of MSK1 was increased in pSS cDC2s compared to HC cDC2s (Figure 3C). Thus, our data suggest that due to their decreased expression of miR-130a, pSS cDC2s have increased expression of MSK1.

### Increased Fraction of IL-12 and TNF-α-producing cDC2s in pSS

**Figure 4:**
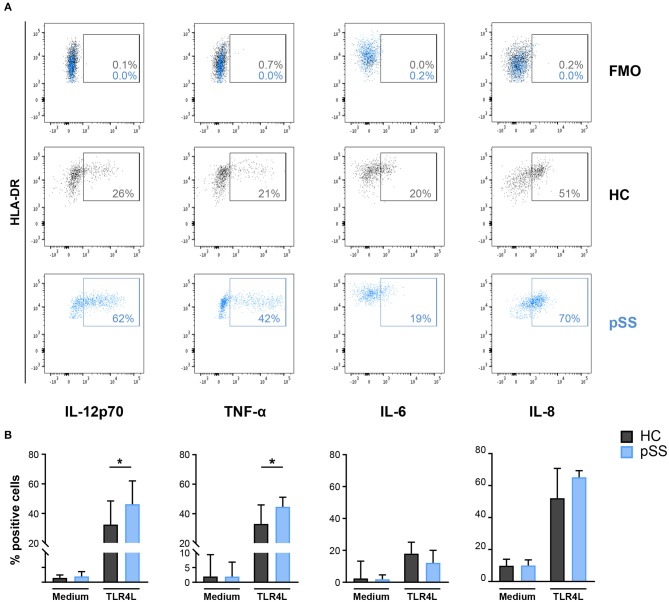
Fraction of cDC2s that produce IL-12 and TNF-α is increased in pSS patients. Intracellular cytokine production was assessed in cDC2s after whole blood stimulation by FACS. Representative flow cytometry dot plots of IL-12, TNF-α, IL-6, and IL-8 expression by cDC2s in HCs (black) and pSS patients (blue) as well as fluorescence minus one (FMO) control upon TLR4L stimulation (25μg/mL) for 6 h are depicted **(A)**. Percentages of cDC2s producing cytokines in resting conditions (medium) or after stimulation (TLR4L) are shown for HC and pSS patients (HC *n* = 12, pSS *n* = 13) **(B)**. Results are represented as median ± IQR. *p < 0.05.

As MSK1 regulates cytokine production and its expression was increased in pSS cDC2s, we next assessed whether cDC2s from pSS patients produce enhanced levels of pro-inflammatory cytokines downstream from MSK1. As activation via TLR4 efficiently induces MSK1 activation (31), this trigger was used as a model to study MSK1 induction in the context of cDC2 activation. Whole-blood from pSS patients and HC was stimulated with TLR4 ligand LPS for 6 h and the production of cytokines by cDC2s was evaluated by flow cytometry. We observed a significantly increased fraction of cDC2s producing IL-12 and TNF-α in pSS patients compared to HC while no significant differences were found for IL-6 or IL-8 (Figures 4A,B).

### MSK1 Blockade Inhibits Pro-inflammatory Cytokine Production by cDC2

**Figure 5:**
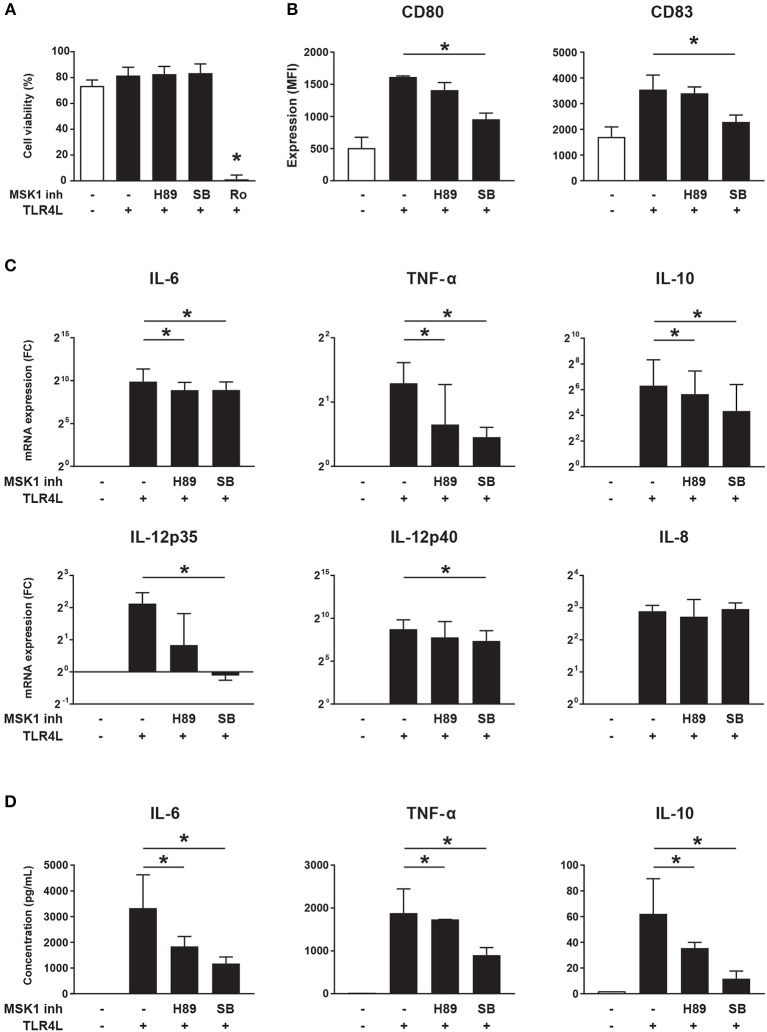
MSK1 inhibition reduces cDC2 activation and inhibits the production of pro-inflammatory cytokines. Isolated cDC2s from buffy coats were treated with H89 (10 μM), SB 747651A (SB; 10 μM), Ro 31-8220 (Ro; 5 μM), or left untreated for 1 h. Then, except for medium control, cells were stimulated with TLR4L (100 ng/mL) for 6 h. FACS was used to assess cell-viability **(A)** and the expression of CD80 and CD83 shown as median fluorescence intensity (MFI) **(B)**. Cytokine production upon TLR4L stimulation in the presence or absence of MSK1 inhibitors was measured by qRT-PCR **(C)** and ELISA **(D)**. Results are represented as median ± IQR. *p < 0.05.

As MSK1 expression is associated with production of pro-inflammatory cytokines (29, 30), we investigated whether MSK1 inhibition can be used to impede cytokine production in cDC2s. For this, we used 3 different inhibitors previously shown to block MSK1 activation (H89, SB 747651A, and Ro 31-8220) (32). The inhibitor Ro 31-8220 was excluded from further analyses due to its significant negative impact on cell viability (Figure 5A). As expected, TLR4 triggering resulted in upregulation of the co-stimulatory molecules CD80 and CD83. Exposure to MSK1 inhibitor SB 747651A lead to clearly reduced expression of both CD80 and CD83, H89 did not affect expression of these molecules (Figure 5B). In addition, we investigated the effects of MSK1 inhibition on the production and secretion of MSK1-dependent cytokines. TLR4 triggering induced expression of IL-6, TNF-α, IL-10, IL-12, and IL-8 on mRNA level. Inhibition of MSK1 signaling with either H89 or SB 747651A before stimulation resulted in a significant reduction of IL-6, TNF-α, and IL-10 mRNA (Figure 5C). Furthermore, the mRNA expression of both IL-12 subunits was decreased upon exposure to SB 747651A, while no effects were observed on IL-8 (Figure 5C). In concordance with the mRNA data, we observed significant inhibition of IL-6, TNF-α, and IL-10 production upon exposure to H89 and SB 747651A at the protein level (Figure 5D); IL-12p70 protein production was not detected (not shown). These data support the targeting of MSK1 to inhibit cDC2s activation and subsequent pro-inflammatory cytokine production.

## Discussion

Using two independent cohorts of patients and controls, we show that the expression of miR-708 and miR-130a is consistently decreased in cDC2s of pSS patients. In addition, these miRNAs are downregulated upon stimulation via TLR3 and TLR7/8. These TLRs have been implicated in the pathogenesis of pSS (27, 28), as they can be triggered by endogenous RNA molecules such as those contained in immune-complexes and are upregulated in salivary gland epithelial cells and PBMCs from pSS patients (33–35). As activation of cDC2s via TLR3 and TLR7/8 leads to downregulation of miR-130a and miR-708 and literature shows that both these miRNAs negatively impact pro-inflammatory cytokine production via regulation of NF-κB signaling (36, 37), these miRNAs seem to have an immune-inhibitory role. In concordance with this, we show for the first time that MSK1 is an endogenous target of miR-130a in primary cDC2s, and that this regulatory axis is altered in cDC2s from pSS patients. Our data indicate that MSK1 is a link between miR-130a and cytokine production, as MSK1 activation leads to the stimulation of NF-κB-driven genes including TNF-α, IL-12, and IL-6 (38, 39).

Circulating cDC2s from pSS patients have increased MSK1 expression, supporting the concept that the observed decrease in miR-130a leads to MSK1 upregulation. Using TLR4 triggering as a model for MSK1-driven cDC2 activation, pSS cDC2s produced higher levels of IL-12 and TNF-α, suggesting that the increased expression of MSK1 contributes to their enhanced cytokine production. In addition, MSK1 activation is downstream of several TLRs (31, 40), in-line with the downregulation of miR-130a upon TLR triggering. Thus, pSS cDC2s have decreased expression of miR-130a and increased expression of MSK1, associated with increased production of TNF-α and IL-12. Both of these cytokines are increased in salivary glands and serum of pSS patients (41, 42). High levels of IL-12 promote immune cell infiltration into the salivary glands, which is associated with the presence of autoantibodies in pSS (43). In addition, IL-12 strongly induces T cell proliferation and Th1 polarization, as well as IFN-γ secretion by effector T cells and NK cells (44). TNF-α is a potent pro-inflammatory cytokine and has been implicated in apoptosis of salivary gland epithelial cells, with subsequent release of nuclear autoantigens that contribute to the production of pSS-specific autoantibodies (41, 45). Thus, the demonstrated enhanced activation of pSS cDC2s supports a crucial role for these cells in local immunopathology.

One limitation of this study concerns the differences in clinical and laboratory parameters between the patient groups included. In this regard, the most relevant differences are those in prevalence of auto-antibodies in the two cohorts used for miRNA profiling, as these may have had effect on miRNA replication, preventing the identification of additional dysregulated miRNAs. Still, no statistically significant differences exist between these two cohorts, and all cohorts included largely represent the average pSS patient population as reported in large patient cohorts (46). MSK1 inhibitor SB 747651A prevented the upregulation of co-stimulatory molecules upon TLR4 triggering. In addition, SB 747651A inhibited the production of pro-inflammatory cytokines IL-6, TNF-α, and IL-12 upon TLR4 stimulation. Dissecting the exact contribution of MSK1 inhibition to reduced cytokine production is hampered by a lack of specific inhibitors without off-target effects. Of the available MSK1 inhibitors, SB 747651A is the most selective and only has minor off-target activity against PKA, RSK, PKB, and S6K (32). Though we cannot rule out off-target effects of SB 747651A, the effects we observe seem to be largely MSK1-specific. First, inhibition with H89, which more strongly inhibits activity of PKA rather than MSK1, showed less inhibition of cytokine production compared to SB 747651A, suggesting that PKA targeting would not lead to the observed effects. Second, specific inhibition of S6K and PKB activity in DCs was previously shown to result in increased IL-12 production and no changes in TNF-α and IL-6 production, which contradicts our results with SB 747651A (47, 48). Third, our data are consistent with previous studies on MSK1-inhibition: exposure of mouse bone marrow-derived DCs to an MSK1 inhibitor (32) as well as silencing of MSK1 in human keratinocytes lead to reduction of pro-inflammatory cytokine production (49). Finally, the effects of SB 747651A on murine macrophage cytokine production closely resembled the phenotype observed in macrophages of MSK1/2 knockout mice (32). Taken together, our data suggest that MSK1 is an important player in cytokine production by cDC2s and its inhibition limits the pro-inflammatory phenotype of these cells.

Thus, using two independent cohorts of patients and controls we provide the first evidence of molecular dysregulation of cDC2s in pSS, including the decreased expression of miR-708 and miR-130a that is associated with TLR-activation. In addition, we show that miR-130a regulates the expression of MSK1, which is a pharmacologically targetable signaling protein that is overexpressed in pSS cDC2s and associated with enhanced production of pro-inflammatory cytokines. In view of its regulation by miR-130a and its central role in NF-κB signaling, MSK1 inhibition to impede pro-inflammatory cytokine production represents a novel therapeutic avenue for treatment of pSS.

## Data Availability

All relevant data are contained within the manuscript, miRNA profiling data have been deposited in NCBI's Gene Expression Omnibus (GSE) and are accessible through GEO Series accession number GSE132842. All the raw data of this manuscript are available by the authors, without undue reservation, to any qualified researcher.

## Ethics Statement

This study was carried out in accordance with the recommendations of the board of medical ethics committee of the University Medical Center Utrecht with written informed consent from all subjects. All subjects gave written informed consent in accordance with the Declaration of Helsinki. The protocol was approved by the board of medical ethics committee of the University Medical Center Utrecht (METC no. 13-697).

## Author Contributions

AL, JvR, SB, EC, AK, MR, TR, and MH were involved in study conception and design. AL, SB, MW, EC, SH, KvdW-J, BB and MH were involved in acquisition of data. Analysis and interpretation of data was performed by AL, MW, AK, MR, JvR, TR and MH. All authors were involved in drafting the article or revising it critically, and all authors approved the final version of the manuscript to be published.

### Conflict of Interest Statement

The authors declare that the research was conducted in the absence of any commercial or financial relationships that could be construed as a potential conflict of interest.
