# Supplementary material for: MicroRNA-130a Contributes to Type-2 Classical DC-activation in Sjögren's Syndrome by Targeting Mitogen- and Stress-Activated Protein Kinase-1
Source: Front Immunol. 2019 Jun 20;10:1335. doi: 10.3389/fimmu.2019.01335 (PMC6595962; doi:10.3389/fimmu.2019.01335)
Supplement: Supplementary file 1 [file Data_Sheet_1.PDF]

Supplementary Material

1 Supplementary Figures and Tables

1.1 Supplementary Figures

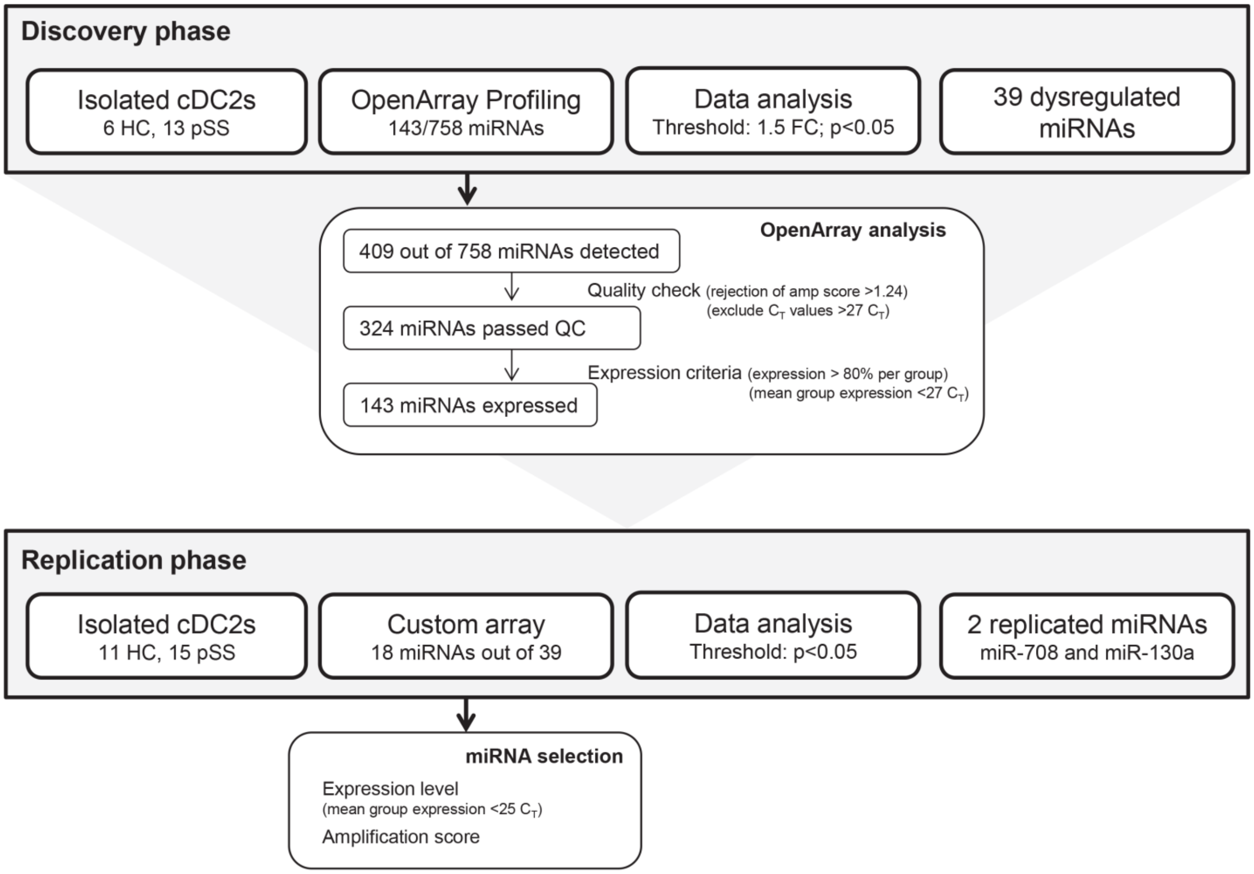

Supplementary Figure 1. Workflow of the miRNA analysis in HC and pSS patients using two independent cohorts

In the replication phase, miRNAs were considered to be replicated when they showed a difference in the same direction (up/down regulated) as in the discovery phase at a p-value <0.05.

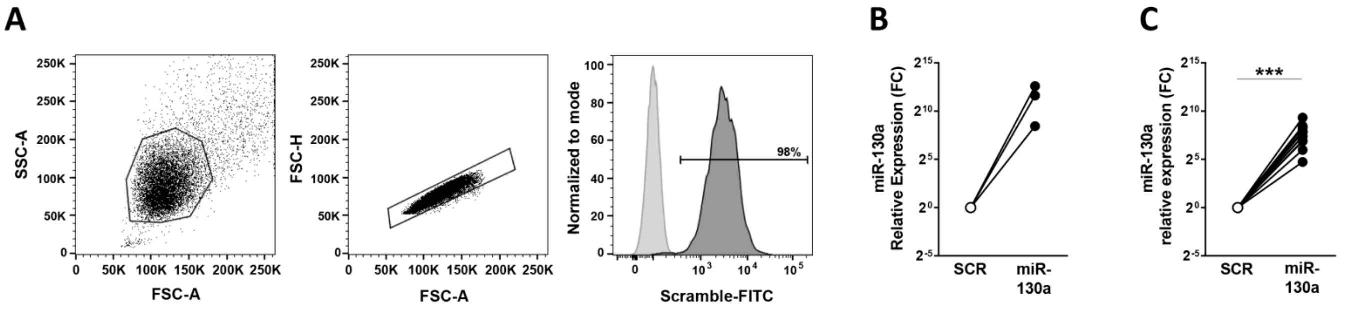

Supplementary Figure 2. miR-130a is efficiently transfected into HEK-293T cells and cDC2s

Transfection efficiency was measured by FACS in HEK-293T cells. The percentage of FITC-positive cells of one representative experiment out of three is depicted (A). miR-130a overexpression was assessed by TaqMan miRNA assays after transfection with miR-130a or non-targeting miRNA control (SCR) both in HEK-293T cells (B) and in cDC2s (C). *** indicates $p \leq 0.001$

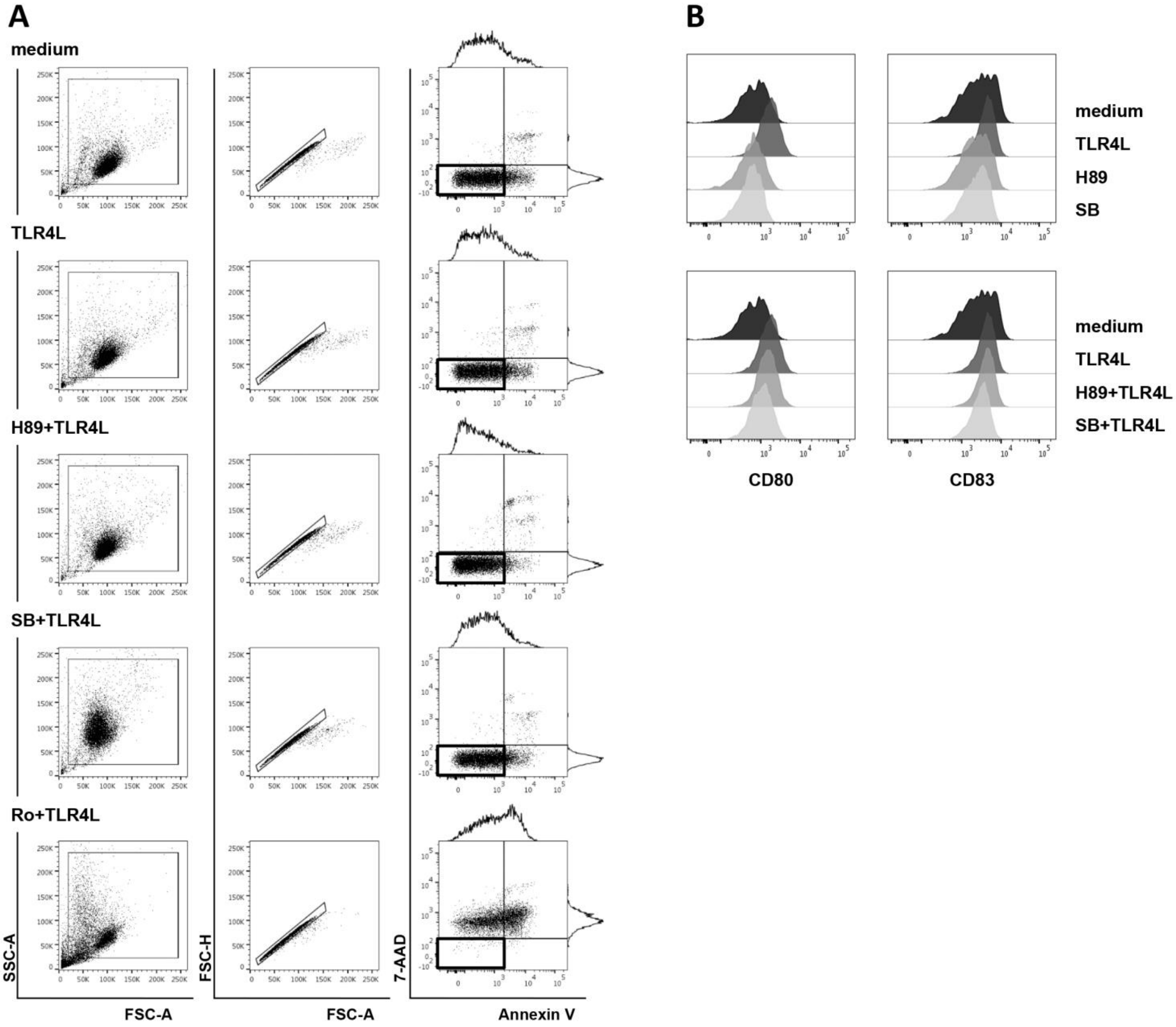

Supplementary Figure 3. MSK1 inhibition with SB 747651A does not affect cDC2 viability and regulates the expression of co-stimulatory molecules.

Gating strategy for analysis of cell viability in cDC2s isolated from buffy coats that were left untreated or were treated either with H89, SB 747651A and Ro 31-8220 for 1h prior to TLR4L stimulation (A). Representative histograms show flow cytometry staining of co-stimulatory molecules in viable (Annexin V and 7-AAD double negative) cDC2s for the different studied conditions (B).

1.2 Supplementary Tables

Supplementary Table 1. miRNAs differentially expressed in both discovery and replication phase.

		Discovery phase		Replication phase	
	**miR**	Fold change pSS vs HC	p-value	Fold change pSS vs HC	p-value
**Downregulated**	**miR-708**	**0.190**	**0.001**	**0.428**	**0.032**
	**miR-142-3p**	**0.243**	**0.028**		
	**miR-30e-3p**	**0.377**	**0.009**	1.012	>0.999
	**miR-378**	**0.390**	**0.005**		
	**miR-213**	**0.398**	**>0.001**	4.666	0.601
	**miR-30d**	**0.403**	**0.031**	0.985	0.919
	**miR-126-5p**	**0.433**	**0.001**	1.611	0.879
	**miR-29b**	**0.434**	**0.018**	1.364	0.919
	**miR-223-5p**	**0.451**	**0.005**		
	**miR-340**	**0.464**	**0.009**		
	**miR-21**	**0.483**	**0.031**	1.105	0.799
	**let-7g**	**0.487**	**0.006**	0.907	0.357
	**miR-25**	**0.495**	**0.006**	0.896	0.377
	**miR-223**	**0.495**	**0.014**	0.946	0.646
	**miR-26b**	**0.506**	**0.009**		
	**miR-140**	**0.514**	**0.047**		
	**miR-345**	**0.520**	**0.007**		
	**miR-150**	**0.529**	**0.011**	0.731	0.799
	**miR-29a**	**0.529**	**0.007**	0.751	0.232
	**miR-26a**	**0.538**	**0.011**		
	**miR-106b**	**0.538**	**0.029**		
	**miR-590-5p**	**0.539**	**0.031**		
	**miR-146a**	**0.541**	**0.033**	1.028	0.959
	**miR-532**	**0.542**	**0.020**	0.953	0.683
	**miR-16**	**0.546**	**0.012**		
	**miR-221**	**0.547**	**0.011**		
	**miR-374-5p**	**0.561**	**0.027**		
	**miR-103**	**0.587**	**0.035**		
	**let-7f**	**0.590**	**0.019**	0.768	0.330
	**miR-130a**	**0.597**	**0.041**	**0.680**	**0.047**
	**miR-629**	**0.610**	**0.040**		
	**miR-335**	**0.616**	**0.012**		
	**let-7e**	**0.622**	**0.015**		
	**let-7d**	**0.625**	**0.032**	0.917	0.721
	**miR-15b**	**0.642**	**0.028**		
	**miR-9**	**0.647**	**0.041**	1.924	0.879
**Upregulated**	**miR-886-5p**	**2.377**	**>0.001**		
	**miR-1248**	**1.719**	**0.041**		
	**miR-1274B**	**1.995**	**0.003**		

Results are expressed as mean FC (p-value). Differences between groups that met the threshold (for discovery: FC difference of 1.5 at p-value of $p<0.05$; for replication: FC difference in same direction as seen in discovery at $p<0.05$) are indicated in bold. Thermofisher Cloud software was used to analyse the data from the discovery phase, while Mann-Whitney U test was used to compare the groups in the replication phase.

Supplementary Table 2. List of the miRNAs targets selected by the prediction algorithms and identified in the proteomic analysis.

	Protein Name	Log2 (Ratio Reverse)	Log2 (Ratio Forward)	Unique/Total Peptides
**miR-708**				
IMPDH1	Inosine Monophosphate Dehydrogenase 1	0.568324	-0.659016	15/17
P4HA1	Prolyl 4-Hydroxylase Subunit Alpha 1	0.504671	-0.458287	5/5
**miR-130a**				
NPTN	Neuroplastin	0.550507	-0.592636	4/4
STX6	Syntaxin-6	0.512075	-0.613035	6/6
ARL6IP1	ADP-ribosylation factor-like protein 6-interacting protein 1	0.500496	-0.360657	5/5
DICER1	Endoribonuclease Dicer	0.475811	-0.731733	18/18
ACSL4	Long-chain-fatty-acid-CoA ligase 4	0.409690	-0.447171	14/17
MSK1	Mitogen- and stress-activated protein kinase-1	0.390998	-0.369334	12/13
RAB5A	Ras-related protein Rab-5A	0.389347	-0.384979	4/8

IMPDH1: Inosine monophosphate dehydrogenase 1; P4HA1: Prolyl 4-hydroxylase subunit α 1; NPTN: Neuroplastin; STX6: Syntaxin-6; ARL6IP1: ADP-ribosylation factor-like protein 6-interacting protein 1; DICER1: Endoribonuclease dicer; ACSL4: Long-chain-fatty-acid-CoA ligase 4; MSK1: Mitogen- and stress-activated protein kinase-1; RAB5A: Ras-related protein Rab-5A.

Supplementary Table 3. Sequences of primers used for RT-qPCR.

	Name	Sequence 5'- 3'
**MSK1 FW**	Mitogen- and stress-activated protein kinase-1	CAACAATCGTTCAAAAGGCCAA
**MSK1 RV**		CGACTGCCTAATGTGTTCCAG
**IL-10 FW**	Interleukin 10	GAGGCTACGGCGCTGTCAT
**IL-10 RV**		CCACGGCCTTGCTCTTGTT
**TNF-α FW**	Tumor necrosis factor alpha	GGAGAAGGGTGACCGACTCA
**TNF-α RV**		CTGCCCAGACTCGGCAA
**IL-6 FW**	Interleukin 6	TGCAATAACCACCCCTGACC
**IL-6 RV**		TGCGCAGAATGAGATGAGTTG
**IL-12p35 FW**	Interleukin 12 subunit p35	CTCCAGAAGGCCAGACAAAC
**IL-12p35 RV**		AATGGTAAACAGGCCTCCACT
**IL-12p40 FW**	Interleukin 12 subunit p40	TGCCGTTCACAAGCTCAAGT
**IL-12p40 RV**		TGGGTCAGGTTTGATGATGTCC
**IL-8 FW**	Interleukin 8	TGAGAGTGGACCACACTGCG
**IL-8 RV**		TCTCCACAACCCTCTGCACC
**RPL32 FW**	Ribosomal protein L32	AGGGTTCGTAGAAGATTCAAGG
**RPL32 RV**		GGAAACATTGTGAGCGATCTC
**ACTB FW**	Actin Beta	CATCGAGCACGGCATCGTCA
**ACTB RV**		TAGCACAGCCTGGATAGCAAC
**B2M FW**	Beta-2-microglobulin	GATGAGTATGCCTGCCGTGT
**B2M RV**		TGCGGCATCTTCAAACCTCC

2 Supplementary Methods

2.1 Stable isotope labelling of amino acids in cell culture (SILAC)

For SILAC labelling, HEK-293T cells were cultured in SILAC-labelled Dulbeccos Modified Eagle Medium (DMEM) (Thermo Fisher Scientific) with 10% dialyzed fetal calf serum (FCS) and 1% penicillin/streptomycin (Thermo Fisher Scientific) containing L-arginine and L-lysine (light medium) or 13C6-L-arginine and 13C6-L-lysine (heavy medium) for at least 14 days to eliminate non-labeled arginine and lysine. The day before the transfection, 1.5x105 cells were seeded in a 6-well plate in a final volume of 3mL. The next day, the medium was changed and the 60-70% confluent cells were transfected with a specific miRNA mimic either for miR-708 and miR-130a or a non-targeting miRNA control (SCR) (Thermo Fisher Scientific) at a final concentration of 30nM together with lipofectamine RNAiMAX and Opti-MEM (Thermo Fisher Scientific). 48h post-transfection cells were harvested by adding lysis buffer containing 8M urea, 1M ammonium bicarbonate, 10nM tris (2-carboxyethyl) phosphine, and 40nM chloroacetamide. Cell lysates were incubated at 95°C for 5 min, sonicated and diluted to 2M urea with 1M ABC. After protein quantification using the BCA protein assay, cell lysates from miR-130a or miR-708-transfected cells generated from heavy medium and SCR-transfected cells from light medium were mixed 1:1 (reverse mode) and vice versa (forward mode). Proteins were digested overnight with 2% (w/w) trypsin, peptides were fractionated based on their molecular mass using ultra performance liquid chromatography (UltiMate-3000 system, Thermo Fisher Scientific), and finally desalted and acidified on a C-18 cartridge (3M). C18-stagetips were activated with methanol, washed with buffer containing 0.5% formic acid in 80% ACN (buffer B) and then with 0.5% formic acid (buffer A). After loading of the digested sample, stage-tips were washed with buffer A and peptides were eluted with buffer B, dried in a SpeedVac, and dissolved in buffer A. Peptides were electro-sprayed directly into an Orbitrap Fusion Tribrid Mass Spectrometer (Thermo Fisher Scientific) and analysed in Top Speed data-dependent mode. Raw files were analysed using Maxquant software. For identification, the human Uniprot was used with both the peptide and the protein false discovery rate set to 1%. Proteins identified were filtered for reverse and decoy hits, standard contaminants and selected to have more than 1 unique or razor peptide by using the Perseus software 1.5.1.6. Heavy/light normalized ratios (ratio reverse and ratio forward) were used to quantify protein expression and were further processed for comparative analysis of differential expression among the conditions.

2.2 Selection of in silico predicted miRNA targets

In silico predicted and experimentally validated targets of the identified miRNAs were retrieved using the miRWalk2.0 database (http://zmf.umm.uni-heidelberg.de/apps/zmf/mirwalk2/) to search in the miRANDA, PICTAR2, PITA, RNA22, miRDB and Targetscan databases. The presence of the miRNA seed-sequence in the target sequence was required. Targets were selected if present in 4 out of the 6 selected databases.

2.3 Quantitative real-time PCR

First-strand cDNA was synthesized from total RNA using Superscript IV kit (Thermo Fisher Scientific), and quantitative real-time PCR was performed on the QuantStudio 12k flex System (Life-Technologies), following manufacturer's instructions. Sequences of the primers used are listed in Supplementary Table 3. RT-qPCR data were normalized to the expression of the selected housekeeping gene and analysed using the comparative CT method as described before.

2.4 Cytokine analysis

Cytokines in cell-free supernatant were measured using enzyme-linked immunosorbent assay for IL-6 (#M9316, Sanquin), TNF-α (#851.570.020, Diaclone), IL-10 (#88-7106-88) and IL-12p70 (#88-7126) (both Thermo Fisher Scientific), following the manufacturer's instructions.
